# Allergen immunotherapy in China

**DOI:** 10.3389/falgy.2023.1324844

**Published:** 2024-01-08

**Authors:** Yaqi Yang, Wenjing Li, Rongfei Zhu

**Affiliations:** ^1^ Department of Allergy, Tongji Hospital, Tongji Medical College of Huazhong University of Science and Technology, Wuhan, China; ^2^ Institute of Allergy and Clinical Immunology, Tongji Hospital, Tongji Medical College of Huazhong University of Science and Technology, Wuhan, China

**Keywords:** allergen immunotherapy, efficacy, safety, allergen extracts, adjuvant

## Abstract

Allergen immunotherapy (AIT) is an etiological treatment strategy that involves administering escalating doses of clinically relevant allergens to desensitize the immune system. It has shown encouraging results in reducing allergy symptoms and enhancing patients' quality of life. In this review, we offer a thorough overview of AIT in China, examining its efficacy, safety, current practices, and prospects. We further underscore the progress made in AIT research and clinical applications, as well as the distinct challenges and opportunities that China faces in this area.

## Introduction

Allergic diseases have become a significant global health concern, with a growing number of individuals being affected by various allergic conditions (1). In recent decades, the prevalence of allergic diseases in China has consistently been on the rise. A study conductedas part of the EuroPrevall-INCO project in China revealed that urban children have higher prevalence rates of self-reported allergic diseases compared to their rural counterparts. Specifically, the study found higher rates of allergic rhinitis (AR) (23.2% vs. 5.3%), asthma (6.6% vs. 2.5%), and eczema (34.1% vs. 25.9%) among urban children (2). A multi-center epidemiological survey conducted in 2011 revealed that the prevalence of adult AR was 17.6% in 18 major cities across China (3). Additionally, a nationwide cross-sectional study reported an adult asthma prevalence in China of 4.2% (4). According to the Global Burden of Disease Study 2019, there was a substantial increase of 25.65% in the number of patients diagnosed with atopic dermatitis (AD) between 1990 and 2019. The prevalence of AD in China has been observed to increase at a faster rate than the global average (5). In the Jiangxi province, around 4% of the adult population reported experiencing food allergies (6). Notably, the prevalence of this condition among children experienced a significant increase, rising from 3.5% in 1999 to 11.1% in 2019 (7). Allergic diseases significantly impact the overall well-being and quality of life of individuals affected by them, as well as their families. Allergen avoidance plays a crucial role in managing allergic diseases. However, it is often challenging to completely avoid these triggers, and conventional pharmacological interventions consistently fall short in meeting the patient's need for symptom relief.

Allergen immunotherapy (AIT) can induce immune tolerance towards allergens, exert a disease-modifying effect on immunoglobulin E (IgE)-mediated allergic diseases, and potentially influence the natural progression of allergic diseases (8, 9). The World Allergy Organization (WAO) suggests that AIT may be considered the only etiological treatment for IgE-mediated allergic diseases (10). Over the years, AIT, including subcutaneous immunotherapy (SCIT) and sublingual immunotherapy (SLIT), has proven to be an effective treatment for patients with AR, allergic conjunctivitis, and allergic asthma due to inhaled allergens (11, 12). SCIT can be administered to individuals diagnosed with Hymenoptera venom allergy (13). Oral immunotherapy (OIT) has emerged as a promising treatment for IgE-mediated food allergy. Several studies have suggested that OIT could be a safe and effective approach for managing peanut, cow's milk, and hen's egg allergies (14).

In China, the use of AIT for the managing allergic conditions began in 1956. Since then, there has been a significant rise in the number of allergic patients in China, accompanied by increased awareness and attention from Chinese doctors towards allergic diseases. Consequently, AIT has gained considerable popularity and has been extensively researched and utilized. In this review, we explore the historical background of AIT in China and summarize its efficacy, safety, current implementation, and future prospects.

## Brief history of AIT in China

In 1956, the establishment of the first Department of Allergy at Peking Union Medical College Hospital (PUMCH) signaled the formal introduction of allergology in China. This development facilitated the provision of medical care to patients affected by allergic diseases. Initially, medical practitioners faced a significant influx of individuals presenting typical symptoms of hay fever. However, the skin prick test (SPT) conducted using allergen preparations procured from American vendors, yielded negative results. This observation prompted doctors to consider that the allergens causing hay fever in Chinese patients might differ from those abroad. Following several years of extensive investigation, Ye and his colleagues identified *Artemisia* pollen and *Humulus* pollen as the primary allergenic pollens in North China (15, 16). From then on, the pioneering group of Chinese allergists developed a comprehensive array of nearly 100 in-house allergen extracts specifically designed for SPT and AIT, catering to the unique characteristics of domestic individuals suffering from allergies. However, all the allergen extracts employed in the study were in the form of crude extracts, including house dust mites (HDMs), pollens, fungi, animal dander, and insect extracts.

After 2001, the National Medical Products Administration (NMPA) undertook efforts to enhance the regulation of allergen preparations, leading to restrictions on the use of in-house crude allergen extracts in certain medical facilities. Currently, the NMPA in China has approved only three standardized allergen extracts of dust mites for AIT. Novo Helisen-Depot (Allergopharma Joachim Ganzer KG, Germany) received NMPA approval in 1999, making it one of the first commercial products available. Alutard-SQ (ALK-Abelló, Denmark), which received NMPA approval in 2004, has gained substantial popularity for SCIT in China. Challergen-Dermatophagoides farinae Drops (Wolwo Bio-Pharmaceutical China) is the only HDM-SLIT product that received approval from the China Food and Drug Administration (CFDA) in 2006 (17). In 2012, the Beijing Municipal Medical Products Administration approved the commercial use of nine types of in-house crude allergen extracts (including dust mite, *Artemisia*, *Humulus*, *Oleaceae*, *Cypress*, *Alternaria*, cat, and dog dander) provided by PUMCH in Beijing and several provinces. In 2021, China approved the marketing of the first standardized drops of Artemisia annua (Wolwo Bio-Pharmaceutical China). In January 2023, thanks to the licensed healthcare policy of Boao LeCheng Medical Advance Zone in Hainan, HDM allergen sublingual tablets (ACARIZAX®, ALK-Abelló, Denmark) were introduced at Ruijin Hainan Hospital in LeCheng, with the first prescription issued on January 13th. The NMPA formally accepted the Biologics Listing License Application (BLA) for ACARIZAX® in 2023. Review and approval are anticipated to be completed in 2024.

## Allergen sensitization profile in China

Inhalant allergens primarily contribute to the etiology of AR and asthma. Similar to other countries, HDMs, such as *Dermatophagoides pteronyssinus* (*Der p*) and *Dermatophagoides farina* (*Der f*), are the main indoor allergens in China (18–20). However, the complex geographical features, diverse climate patterns, and varied levels of industrial development across China have led to significant variations in allergenic substances across different regions. In China, there is a noted decreasing trend of sensitization to HDM from the southeast to the northwest region (20). For instance, in central China, HDM was the dominant allergen, with a sensitization rate exceeding 90% (18). In contrast, in Lhasa, HDM was ranked as the fourth most common inhalant allergen (21). Overall, the prevalence of sensitization to HDM in China continues to rise (22).

Pollen allergens display regional variations influenced by the dominant plant species in different geographical locations. *Artemisia* pollen is widely recognized as the leading allergen in the northern region of the Yangtze River in China (20). From 2008 to 2018, there was a significant increase in the prevalence of pollen sensitization, particularly to *Artemisia vulgaris*, in the northern region of China (22). During a study conducted in Central China, it was noted that more patients exhibited sensitization to tree pollens, specifically *Platanus*, compared to *Artemisia* (18).

Sensitization to animal allergens has seen a notable increase, especially among children and adolescents. Cat dander is the most common allergen of animal origin (23). In Central China, there has been a significant rise in the overall positive rates of cat and dog dander from 1.3% to 15.5% and 0.8% to 10.5%, respectively, between 2016 and 2021 (24).

Fungi are also significant inhalant allergens in China. The prevalence of mold sensitization was reported to be higher in children diagnosed with rhinitis and asthma (22). In a study conducted in Wuhan, SPT data from 1,365 patients with respiratory allergies revealed that 14.8% of them exhibited sensitization to fungi. The most commonly detected fungi allergens were *Cladosporium* (11.72%), *Penicillium* (4.76%), and *Alternaria* (4.69%) (25). Similar to other allergens, there has been an observed increase in sensitization rates to *Cladosporium* and *Alternaria* over the past five years (24). In Taiwan, a study conducted revealed that the fungal species most likely to induce allergic reactions were *Candida*, *Aspergillus*, and *Penicillium* (26).

## Allergen sensitization profile in other countries

Asian countries, with their diverse climate conditions, foster a variety of plants and animals. In Asia, Japanese cedar (27) and Japanese hop (28) are the two most distinct sources of pollen allergens. Oak (29), mugwort, and grass are also common, but their species differ from those found in Western countries (30).

A multicenter study conducted across 14 European countries revealed that grass pollen, HDM, birch pollen, cat dander, olive pollen, mugwort, German cockroach, and *Alternaria* are the most common allergens in the majority of subjects across these countries (31). Allergy sensitization in Southern Europe is relatively straightforward, with grass and olive allergies being the most common throughout most of the region. Conversely, Northern and Central Europe present a more complex sensitization profile, with allergies to grasses and birch pollen being dominant. Moving towards Central and especially Eastern Europe, allergies to *Ambrosia*, *Artemisia*, and ash tree pollen may appear (9).

In the United States (US), the population is most commonly sensitized to grass pollen, dust mites, and ragweed pollen (32). The sensitization rate to fungi in the US ranges from 7.4% to 18.6% with the highest rates for *Candida albicans* (18.6%), *Alternaria alternata* (16.6%), *Stemphylium herbarum* (14.9%), and *Aspergillus fumigatus* (14.2%) (33).

## AIT in China

### Efficacy of SCIT

In China, numerous non-standardized crude extracts have been employed for a long duration and have demonstrated significant efficacy (34–40). In 1987, a one-year controlled trial was carried out to evaluate the efficacy of SCIT in 50 patients with hay fever who were sensitive to *Artemisia*. The trial results showed a significant improvement in symptoms among the treatment group, with an overall effective rate of 78% (34). A cross-sectional, real-world study was conducted at multiple centers to assess the effects of SCIT using allergen crude extracts on 246 patients with AR, with or without asthma. The study found that 96.7% of the patients experienced an improvement in clinical symptoms following SCIT. Additionally, the use of concomitant medications, such as antihistamines and nasal corticosteroids was reduced after SCIT (35). Du et al. conducted a retrospective evaluation to assess the effectiveness and safety of SCIT using mixed allergens for the treatment of allergic asthma (36). The study revealed an increase in the percentage of forced expiratory volume in the first second to the predicted value (FEV1%) in all patients, in addition to symptom relief and a reduction in concomitant medication. Furthermore, a retrospective study spanning three years and involving a total of 1,640 patients was conducted to examine the impact of SCIT on the occurrence of new sensitization in individuals with respiratory allergies (37). In this study, the crude allergen extracts utilized included dust mites, weed pollens, grass pollens, molds, and animal dander. The study revealed that patients who were mono-sensitized had a lower likelihood of developing new sensitization compared to those who were multi-sensitized. New sensitization is observed during the initial phases of SCIT, with a subsequent decline in the rate of new sensitization over time.

Standardized HDM extracts have been utilized in SCIT for patients with AR and asthma in China for over 20 years. The utilization of these extracts has consistently demonstrated favorable efficacy (41–68).

In a historical cohort study conducted in Guangzhou, a total of 158 patients with persistent AR were included. Out of these, 114 patients received treatment with a standardized mite depot-allergen extract (NovoHelisen Depot, Germany), which consisted of a 50% mixture of *Der p* and *Der f* allergens (59). This study presented findings that support the effectiveness of SCIT in treating patients with AR caused by HDM allergens. The study utilized a standardized allergen product and observed improvements in clinical symptoms both after the termination of SCIT and during the 2-year follow-up period. Importantly, SCIT demonstrated a significant reduction in the likelihood of asthma development among patients with AR even after discontinuation of SCIT for 2 years. Furthermore, there was a head-to-head study that compared the efficacy and safety of *Der p* extracts (Alutard SQ) and *Der p/Der f* extracts (Novo Helisen Depot) in AR patients (60). This study has confirmed that both HDM extracts exhibit equal efficacy and safety profiles. A randomized trial conducted in Mainland China aimed to investigate the efficacy of SCIT with Alutard-SQ in mild to moderate allergic asthma. The study involved 132 asthmatic patients aged 6–45 years, who were recruited from three different regions of Mainland China. This study was the first of its kind in China and employed a double-blind, placebo-controlled design (61). The findings indicated that the scores for symptoms started to decrease at week 29 and persisted until week 48 in the immunotherapy group. They also observed a decrease in SPT response in the immunotherapy group, while the levels of *Der p*-sIgE remained unchanged. Another study, which followed a randomized, double-blind, placebo-controlled design, examined the effectiveness of SCIT with Alutard-SQ over three years. The study included a sample of 90 children diagnosed with AR and asthma (62). The findings indicate that a 3-year course of SCIT has the potential to decrease both day-time and night-time asthmatic symptom scores, improve peak expiratory flow (PEF) values, lower serum IgE levels, and most notably, reduce the requirement of inhaled corticosteroids (ICSs). Zhang et al. conducted a study in which they recruited 51 children with allergic asthma to compare the effectiveness and safety of long-term HDM-SCIT in mono- and polysensitized children (63). In terms of clinical effectiveness and safety of HDM-SCIT, there was no significant difference between mono-sensitized and poly-sensitized children with allergic asthma. However, Huang et al. conducted a comparative analysis to assess the effectiveness and safety of HDM-SCIT in patients with HDM mono-sensitized AR and individuals sensitized to multiple allergens. Their study yielded contrasting outcomes (64). The findings suggested that HDM-SCIT was safe and effective for AR patients with only HDM and multiple allergens including HDM, but the profile of allergen sensitization could potentially influence the efficacy of SCIT, notably, the efficacy of SCIT was more pronounced in AR patients who were sensitized to three or fewer allergens, excluding HDM. The difference in the results of the two studies may be related to the selected diseases, sample size, and there were fewer patients with pollen allergy in Zhang's trial.

Overall, numerous studies provide evidence supporting the effectiveness of SCIT in the management of AR and/or asthma among Chinese patients. These studies have primarily concentrated on the following areas: (1) the alleviation of allergic symptoms and enhancement of quality of life, (2) the reduction of drugs used for symptomatic treatment, (3) the durability of effects even after the cessation of treatment, (4) the prevention of AR from progressing into asthma, (5) the prevention of new sensitization.

Furthermore, Zhou et al. conducted a retrospective analysis to evaluate the long-term effectiveness and safety of SCIT in patients with AD who were sensitized to HDM (69). In this study, a total of 164 patients were administered SCIT plus pharmacotherapy for 3 years. Additionally, a separate group of 214 patients with AD solely received pharmacotherapy. The findings of this study revealed a significant decrease in both the symptoms of AD and the scores on the pruritus visual analog scale (VAS) in the SCIT group compared to the non-SCIT group. This decrease was observed after 3 years of treatment.

### Safety of SCIT

The safety profile of SCIT has been demonstrated to be favorable in both adults and children, as evidenced by data from Randomized Controlled Trials (RCTs) and clinical practice in China (70–78).

Wen et al. conducted a review of adverse events (AEs) observed in patients who underwent SCIT using crude pollen allergen extracts at their department between December 1993 and September 2013 (70). A total of 70 AEs were observed in 35 patients. The study found that a significant proportion (97.1% or 68/70)) of systemic reactions (SRs) occurred when the maximal concentration was administered. Among these SRs, the majority were classified as mild to moderate, with 58.6% being grade 1, 15.7% grade 2, 17.1% grade 3, and 8.6% grade 4. Several risk factors were identified, including the administration of large doses (0.6–1.0 ml), increasing doses during the pollen season, administering higher doses without considering obvious local reactions (LRs), and suspected incorrect injection techniques.

Yang et al. conducted a study to investigate the safety of HDM-SCIT (Alutard SQ, ALK) in preschool children diagnosed with respiratory allergic diseases (71). A total of 3,109 injections were recorded in 91 patients. Out of these injections, 186 (5.98%) resulted in immediate LRs in 62 (68.13%) patients. Additionally, 6 injections (0.19%) led to delayed LRs in 4 patients (4.4%), while 44 injections (1.42%) caused immediate SRs in 11 patients (12.09%). This study revealed that body mass index (BMI) and HDM-sIgE were identified as risk factors for LRs. A multicenter study was conducted to investigate the safety of semi-depot HDM allergen extract (Novo-Helisen Depot) in children and adolescents diagnosed with AR and asthma (74). A total of 3,600 injections were administered to 250 patients. Among these injections, 361 (10%) were associated with SCIT-related AEs occurred in 96 (38.4%) of the patients. Additionally, 321 injections (8.9%) resulted in LRs occurring in 89 (35.6%) patients, while 40 injections (1.1%) led to SRs occurring in 23 (9.2%) patients.

## SLIT

### Efficacy of SLIT

Numerous studies have demonstrated the short- and long-term efficacy of SLIT in AR and/or asthma in both adult and pediatric patients in China (79–98). Individualized treatment is essential to improve response rates to SLIT, as there are variations in efficacy and side effects among individuals. Gao et al. enrolled 157 AR patients aged 4–60 years, and categorized patients into high response (HR) and low response (LR) groups based on reductions in combined symptom and medication scores (CSMS) after 6 months of SLIT treatment (83). HR groups were the patients with CSMS reduced by over 50% and continued the original dose, while the LR groups were the patients with CSMS down 20%–50% and received an increased dose (the percentage of dose increase was 33.33% for patients younger than 14 years of age and 50% for patients older than 14 years of age). They found a significant difference in CSMS and VAS between the two groups at 6 months and 1 year, but not in later follow-ups. They concluded that dosage enhancement within a certain range may improve the efficacy of SLIT.

SLIT of *Artemisia annua* is currently being conducted in China and has shown promising efficacy (34, 99–103). A randomized, double-blind, placebo-controlled phase 3 clinical trial involving 71 seasonal AR investigated the efficacy and mechanisms underlying SLIT of *Artemisia annua* (100). The results revealed that SLIT with *Artemisia annua* consistently improved patients' nasal symptom scores during peak pollen season in years one and two, decreased Th2 cells, increased nTreg and Tr1 cells in blood after 16 weeks, increased Cystatin 1 in nasal secretion after 16 and 32 weeks. Another randomized, double-blind, placebo-controlled, multicenter, phase III clinical trial was conducted to assess the efficacy and safety of SLIT in 702 patients with *Artemisia annua*-induced AR (101). The findings of this study indicate that SLIT had a significant positive impact on the severity of rhino-conjunctivitis and total nasal symptoms experienced daily. Additionally, SLIT was found to effectively reduce the need for daily rescue medication during the peak pollen period. Yang et al. conducted a study to investigate the efficacy and safety of *Artemisia annua*-SLIT in seasonal AR patients, focusing on the impact of different intervention times (102). This study has provided evidence to support the equivalent efficacy and safety of *Artemisia annua*-SLIT in the treatment of seasonal AR patients. The study found that both 8–9 and 12–13 weeks of pre-season therapy with *Artemisia annua*-SLIT resulted in comparable outcomes in terms of efficacy and safety. This was observed in both mono-sensitized and poly-sensitized groups. The investigation of the long-term efficacy and safety of *Artemisia annua*-SLIT is necessary.

The SLIT has also demonstrated effectiveness in treating AD (104–106). A multicenter, randomized, double-blind, placebo-controlled clinical trial was conducted over 36 weeks. The trial involved 239 patients diagnosed with AD and aimed to evaluate the efficacy and safety of SLIT using *Der f* Drops (105). This study reported that significant decreases in Scoring Atopic Dermatitis (SCORAD) indexes, skin lesion area scores, dermatology life quality indexes, and total medication scores were seen in both the medium- and high-dose groups.

### Safety of SLIT

Both SLIT with *Der f* drops and SLIT with *Artemisia annua* drops have demonstrated a satisfactory safety profile in both children and adults (93, 100–102, 107–110).

Shao et al. conducted a study that aimed to investigate the effectiveness and safety of SLIT in young children (264 children aged 3–13 years old, including 133 children aged 3–5 years old) (93). There were no significant differences in clinical efficacy, time to onset, immunologic parameters, or safety between children younger and older than 5 years of age in the SLIT group. No serious systemic AEs were reported.

In the study of *Artemisia annua*-SLIT, Lou, et al. reported that 17/47 patients experienced mild local AEs and 2 patients experienced mild systemic AEs. The most common AEs observed were oral paresthesia, nasopharyngitis, sneezing, nasal pruritus, rhinorrhea, eye pruritus, nasal congestion, throat irritation, oropharyngeal pain, cough, upper respiratory tract infection, ear pruritus, headache, throat-clearing, diarrhea, tongue itching, and swollen tongue, listed in the descending order of frequency (100). In a multicenter randomized trial on *Artemisia annua*-SLIT trial (101), no serious SLIT-related AEs were reported.

### SCIT vs. SLIT

After many years of clinical practice, both SCIT and SLIT have exhibited favorable outcomes. In general, SLIT has been observed to have fewer and milder adverse effects compared to SCIT, while SCIT typically demonstrates greater effectiveness and has a faster onset of action (17, 111–115). A prospective, open-label, and single-center study was conducted to compare the efficacy, safety, and compliance of SCIT and SLIT in HDM-induced AR children (116). Their results suggested SCIT had a higher compliance rate than SLIT, whereas SLIT had fewer adverse events than SCIT. The total nasal symptom score, rescue medication score, and symptom medication score were all lower in the SCIT group than that in the SLIT group. However, in other studies SLIT has same clinical effect compared with SCIT (117–121). Xian et al. compared clinical effectiveness and immune responses between SLIT and SCIT in AR sensitized to HDM (120). They found that both SLIT and SCIT have similar rates of clinical improvement. In both groups, there was a trend towards upregulation of CD4 + CD25 + FoxP3+ Tregs, but this was only found to be inversely correlated with total rhinitis score in SLIT. Furthermore, the levels of *Der p* specific immunoglobulin G4 (*Der-p*-sIgG4) increased significantly in both SCIT and SLIT group, but it was found to be 30 times higher in SCIT than SLIT after the treatment.

### AIT in other countries

The efficacy and safety of AIT for AR and asthma have been confirmed in other Asian countries (122–129). The effect of AIT on AD has also been reported (130–133).

In Korea, the commercial allergens used for SCIT include HDM, pollens, mold and animal epithelia, while SLIT was prescribed only for HDM (134). SCIT prescription is more popular than SLIT in Korea (125).

In Japan, SCIT was introduced in the early 1960s as a treatment for AR and/or asthma. Nowadays, SCIT and SLIT are both permitted for patients allergic to Japanes Cedar Pollen (JCP) and HDM (135–137), and dual SLIT for JCP and HDM is also safe (138). SLIT in form of liquid formulations and tablets are both available in Japan (137). Sales of SCIT products in Japan declined steadily from the 1980s, possibly related to the disadvantages of SCIT (137). The first Japanese cedar SLIT drop product, Cedartolen, was registered in October 2014, and has been shown to significantly reduce the total nasal symptom and medication scores in Phase II and III clinical trials (139, 140). HDM SLIT tablets (Miticure and Actair) were launched in 2015 (141–143). A JCP SLIT tablet was developed in 2018 based on the same efficient freeze-dried formulation as Miticure (144). This JCP SLIT tablet now approved for market in Japan as named Cedarcure.

In the US, there are currently 4 companies that manufacture and market allergen extracts for clinical use, and allow standardization of 19 SCIT products for HDM, molds, pollens, Hymenoptera venom, mammalian epithelia and feathers, whole body insect and miscellaneous items. US Food and Drug Administration (FDA) approved tablet products standardized for allergenic potency include grass, ragweed, HDM and a grass mix. However, it's worth noting that most commercially available allergen extracts are not standardized (145, 146).

In Europe, the first SCIT products were authorized in 1976, whereas the first SLIT product was authorized in Germany in 2004, and most currently available SCIT products were authorized in the 1990s, more product options are available in Europe, including adsorbed allergens, chemically modified allergens, or both. Both tablets and liquid extracts are approved for SLIT (9). There are major differences in the clinical approach to SCIT in polysensitized patients, European allergists suggested preferably do not mix more than 3 components in a single vaccine, whereas in US mixed extracts containing multiple aeroallergens are used (145).

## Modified regimen and novel routes of administration of AIT

### Rush and cluster immunotherapy schedules

Rush immunotherapy (RIT) offers the most expedited build-up time, reducing up-dosing treatment from the traditional 4 months to less than 1 week. A prospective, open-label phase IV clinical trial compared the efficacy and safety of RIT and conventional immunotherapy in AR patients (147). The study showed a reduced incidence of adverse effects and a decreasing trend in leukotriene levels among both the RIT and conventional immunotherapy groups, suggesting comparable safety profiles. Notably, the VAS scores showed a decrease in the RIT group at the end of the second week. Additionally, the levels of IgG4 were found to be higher after the completion of the RIT dose accrual, one week later. Moreover, the weekly drug dosage scores were comparatively higher in the conventional immunotherapy group, suggesting that RIT exhibits a more accelerated onset of action when compared to conventional immunotherapy.

Cluster immunotherapy typically takes 4–8 weeks to reach a maintenance dose and requires patients to receive multiple allergen injections (generally two to four injections) sequentially in a single day of treatment on non-consecutive days. A randomized and open-label trial enrolled 149 AR patients to compare the efficacy of conventional and cluster immunotherapy during the build-up phase in adults and children (148). After the completion of the build-up phase of immunotherapy, a significant decrease in symptom scores was observed among the majority of patients, regardless of whether they followed the conventional or accelerated cluster schedules. However, there was no difference in efficacy between conventional and cluster SCIT, nor between adults and children. Similar results have demonstrated the safety of RIT and cluster immunotherapy in other studies (149–160).

### Intralymphatic immunotherapy (ILIT)

ILIT is a novel route of immunotherapy for patients. Several studies on ILIT in China have shown promising results (161–165). A pilot study was conducted to assess the clinical effectiveness and safety of cervical ILIT in HDM-induced AR adult patients (163). This trial demonstrated that ILIT significantly improved both symptoms and quality of life, reduced administration of rescue medication, and no moderate or severe adverse events. Another prospective randomized controlled trial, spanning over 3 years, assessed the long-term efficacy and safety of cervical ILIT in 50 children with HDM-induced allergic rhino-conjunctivitis (164). The trial showed that compared with SCIT, cervical ILIT could improve allergic symptoms more rapidly, shorten the period of treatment, and lower pain perception. However, the long-term effects were found to be better in the SCIT group. The cervical ILIT group was safer, as evidenced by the occurrence of only 3 mild local adverse reactions and the absence of any systemic adverse reactions in the cervical ILIT group. In contrast, the SCIT group experienced 14 systemic adverse reactions. Wang et al. conducted a study on adult AR patients to evaluate the long-term effectiveness of cervical ILIT (165). They found that the cervical ILIT had long-term efficacy, high safety, and high compliance, but its long-term efficacy was inferior to that in the SCIT group.

### Epicutaneous immunotherapy (EPIT)

In China, there have been no clinical studies of EPIT, but several studies in mice model have reported its efficacy and safety (166, 167). Zhang et al. used composite microneedles (MNs) to deliver sustained antigens for EPIT (167). They found that this novel EPIT is more effective at a lower dose compared to conventional SCIT. However, clinical data is needed to demonstrate its efficacy and safety before it can be approved for routine clinical use.

## Biologicals in AIT

In recent years, there has been a significant increase in the inclusion of biologicals in medical insurance, leading to their widespread utilization in China. Several studies have substantiated the efficacy of omalizumab in RIT (168–171) or cluster immunotherapy (172). For instance, Zhang et al. designed a real-world retrospective study to investigate the efficacy, safety, compliance, and cost of combination treatment with RIT plus one dose of pretreatment omalizumab in Chinese children with respiratory allergies (169). The findings of this study indicate that RIT plus one dose of pretreatment omalizumab had comparable safety, better adherence, and potentially faster onset of efficacy at no additional cost compared to conventional immunotherapy. Huang et al. conducted a comparative study to evaluate the short-term efficacy and safety of conventional SCIT, RIT, and RIT plus one dose of pretreatment omalizumab. The findings revealed that the addition of omalizumab to RIT resulted in a significant improvement in early-stage efficacy. Furthermore, this therapy exhibited the advantages of effectiveness, safety, and convenience (170).

In addition, several studies have shown that omalizumab combined with SCIT can enhance the efficacy and safety of SCIT, while decreasing adverse event (173–178). For instance, Long found that omalizumab combined with SCIT can achieve complete asthma control faster, with a reduction in the amount of asthma medication used, and a better improvement in lung function. Compared with SCIT alone, omalizumab combined with SCIT had a lower incidence of adverse events (174).

Some hospitals are currently implementing a combined treatment approach involving Dupilumab and AIT. Deng et al. retrospectively observed the efficacy and safety of 10 patients with moderate-to-severe AD, treated with a combination of Dupilumab and SCIT (179). This study indicated that dupilumab and SCIT combination therapy was safe and effective for treating moderate to severe AD patients who are resistant to either dupilumab or SCIT monotherapy. However, further clinical research is required to fully understand the role of Dupilumab in AIT.

## Adjuvants

Adjuvants encompass a diverse range of complexes that serve as depot foundations, enhancing the stimulation and modulation of protective responses (180). Several animal studies conducted in China have investigated the use of adjuvants for the treatment of allergic diseases. These studies have demonstrated promising potential for development, as outlined in Table 1. Chitosan is one of the most explored polysaccharide for mucosal vaccine delivery. Li et al. entrapped Der f 2-47-67 in chitosan to obtain Derf 2-47-67 loaded chitosan microparticles, which were injected intraperitoneally into asthma mice (181). The results showed Der f 2-47-67-loaded chitosan microparticle inhibited airway allergic inflammation. Similar to Li et al, Yu et al. prepare Der f chitosan nanoparticle vaccine to treat asthma mice by sublingual administration (182). Der f chitosan nanoparticle vaccine could reduce airway hyperresponsiveness (AHR) and lung inflammation. Xu et al. investigated the immunological adjuvant effect of silver nanoparticles (AgNPs) *in vitro* and vivo (183). The results indicated that AgNPs elicited Th2-biased immune responses *in vivo* and could recruit and active local leukocytes and especially macrophages *in vitro*. Ma et al. prepared a new versatile Toll-like receptor 7 agonist (TLR7a) conjugate to Der f 1 (184). They found the course of AHR and eosinophilia of the TLR7a vaccine-treated mice were limited. The levels of specific IgG1, IgG2a, and IgE antibodies in these mice exhibited significant changes compared to those in the model mice. Following treatment with the TLR7a vaccine, there was a notable decrease in the expression of Th2 cytokine interleukin (IL)-4 production in bronchoalveolar lavage fluid (BALF) and splenocytes, while the levels of interferon-γ (IFN-γ), IL-12, and IL-10 were significantly increased.

**Table 1:** Animal studies of adjuvants in allergic diseases in China (180).

Model of study	Type of adjuvant	Influence on diseases	Reference
Mouse	*Der f* 2-loaded chitosan microparticles	• Reduced AHR • Reduced amounts of eosinophils in BALF • Relieve lung inflammation • Decrease mucus creation • Decreased IgE in serum • Increased IgG2a in serum	Li et al. (181)
Mouse	*Der f*-f-loaded chitosan nanoparticle	• Reduced AHR and lung inflammation • Reduced numbers of total cells and eosinophils in BALF • Reduced sIgE and increased IgA • Regulated the levels of Th2/Th1 cytokines • Inhibited the proliferation of allergen-specific splenocyte	Yu et al. (182)
Mouse	*In vivo* and *in vitro* using of AgNPS by model antigens OVA and BSA	• Reduced IgG production • Reduced Th2 immune responses • Rise of IgG1/IgG2a and IgE • Rise of peritoneal leukocytes I, TNF-α, and IFN-γ	Xu et al. (183)
Mouse	Conjugation of TLR7 agonist to *Der f* 1	• Reduced AHR and airway inflammation • Reduced IgE and increased IgG1 and IgG2a • Regulated the levels of Th2/Th1 cytokines • Suppressed lung inflammation	Ma et al. (184)

AHR, airway hyperresponsiveness; BALF, bronchoalveolar lavage fluid; IFN-γ, interferon-γ; Ig, immunoglobulin; NP, nanoparticle; TNF-α, Tumor necrosis factor-α; TLR, Toll-like receptor.

## DNA vaccines

DNA vaccines are not only immunogenic and safe, but they also offer greater flexibility than previous protein vaccines, as they can be easily modified and constructed. Various DNA vaccines have been studied for their effects in mice in China (185–191). Recently, Hu et al. developed a DNA vaccine that co-expressing *Der p*2 and A20 protein (Pvax1-*Der p*2-A20). This vaccine was encapsulated into poly (L-lactide-co-glycolide) (PLGA) nanoparticles, and its effect was investigated through intranasal administration in mice with AR (191). The results indicated that this DNA vaccine could alleviate nasal allergic inflammation, and inhibit serum *Der p*2-sIgE, IL-4, and IL-13 expression. Concurrently, it increased *Der p*2-sIgG1, IgG2a and IFN-γ expression in serum and splenic CD4^+^CD25^+^Fox3^+^Treg population.

## Recombinant allergens

Lactic acid bacteria (LAB), considered safe for consumption and possessing probiotic properties, have gained attention as potential carriers for mucosal vaccines due to their safety profile and probiotic nature. LAB have also been recognized for their anti-allergic effects. An increasing number of studies have used LAB to express a variety of heterologous antigens as oral vaccines, with several of these studies conducted in China (186, 192–194). Ren et al. used transgenic LAB to produce the peanut allergen *Ara h*2 through various protein-targeting systems and investigated the immune-modulatory efficacy of these systems on allergic immune responses in mice (194). The results demonstrated that oral administration of recombinant LAB could induce sIgA and regulatory T cells at the local levels. Charng et al. designed recombinant LAB containing a plasmid-encoded *Der p*5, and found that these recombinant LAB could suppress allergen-induced airway inflammation (192).

## Summary

Allergic diseases have imposed a substantial burden on public health in China, with a significant increase in the proportion of the population affected by such diseases. This trend has necessitated effective treatment options like AIT. In China, AIT has gained significant recognition and is widely used in clinical practice. While AIT is available in major cities and specialized allergy clinics, its accessibility in rural areas and smaller cities remains limited.

China is actively working towards creating standardized allergen extracts and treatment protocols to ensure quality and efficacy, researching novel allergens, personalized immunotherapy, and adjuvants to enhance the effectiveness of AIT. However, raising awareness among patients and healthcare providers about the benefits and safety of AIT remains a challenge.

In the future, it will be necessary to expand the range of allergen extracts available for immunotherapy to cover a broader spectrum of allergens specific to the Chinese population. Future directions also include personalized immunotherapy, tailored to an individual's specific allergens and immune response. Meanwhile, enhancing the knowledge and skills of healthcare professionals through training programs and continuing medical education can improve the implementation and effectiveness of AIT. Providing support and education to patients, including information about AIT, managing expectations, and addressing concerns, can improve patient adherence and satisfaction. Efforts should be made to increase the accessibility of AIT in rural areas and smaller cities through training programs for healthcare professionals and the establishment of more specialized clinics.

In conclusion, while AIT is already being used in China, further development, standardization, and accessibility are still needed. With advancements in technology and increasing research efforts, AIT has the potential to become a widely available and personalized treatment option for allergic diseases in China.
